# Vertebral fracture caused by electric shock: case report and systematic review

**DOI:** 10.3389/fsurg.2026.1762550

**Published:** 2026-05-18

**Authors:** Dan Liu, Xin Chen, Jingming Wang, Kai Wu, Fangda Geng, Weimin Huang

**Affiliations:** 1 Yunnan Cancer Hospital, The Third Affiliated Hospital of Kunming Medical University, Kunming, Yunnan, China; 2 Department of Orthopedics, 960th Hospital of PLA, Jinan, China; 3 Department of Burns, 960th Hospital of PLA, Jinan, China; 4 Naval Medical University, Shanghai, China

**Keywords:** case report, electric shock, neuropathic pain, systematic review, vertebral fracture

## Abstract

**Objective:**

This study aims to explore the clinical characteristics of spinal fractures resulting from electric shock injuries. By analyzing a rare case of spinal fractures following electric shock, combined with a review of existing literature, we aim to provide clinicians with guidance on the diagnosis and treatment of such injuries.

**Methods:**

We reported a 49-year-old male patient who sustained a spinal fracture due to electric shock, including his medical history, physical examination findings, imaging studies, and clinical prognosis. Additionally, a systematic review of relevant literature was conducted to analyze the mechanisms, diagnosis, treatment, and prognostic characteristics associated with spinal fractures caused by electric shock injuries.

**Results:**

The patient sustained a severe spinal fracture as a result of an electric shock. Imaging examinations indicated the presence of compression fractures at T1 and burst fractures at T4 and T6. Following open reduction, the patient's back pain was significantly alleviated postoperatively. A systematic review encompassed 12 cases, all of which involved male patients with an average age of 36.4 years, ranging from 14 to 50 years. Analysis of fracture distribution revealed that *75%* (9 out of 12) of the compression fractures were localized to the thoracic spine.

**Conclusion:**

All 12 cases included in this systematic review were male patients, which may reflect the higher occupational exposure risk of men to electrical hazards and potential reporting bias. For individuals experiencing back pain following an electric shock injury, particularly male patients, it is essential to consider the potential for spinal fractures to prevent missed diagnoses.

## Introduction

Electrical injuries are a prevalent form of accidental trauma, with the extent of damage to the human body influenced by various factors, including the intensity, type, duration of contact, and individual variations in response to electrical current (1). Clinical manifestations of electrical injuries encompass not only common skin burns but also damage to the nervous system and cardiopulmonary dysfunction; notably, fractures can represent a potentially serious consequence of such injuries (2–6). However, spinal fractures resulting from electrical injuries are relatively uncommon, and existing research on their pathogenesis, clinical characteristics, and treatment strategies remains limited.

Recent advancements in the study of electrical injuries have led to an increasing number of reports documenting fractures associated with electrical trauma, thereby enriching our understanding of this complex phenomenon (7). These studies suggest that the mechanisms underlying fractures resulting from electrical injuries may involve several factors, including the thermal effects of electric current, muscle spasms, and secondary blunt trauma (8, 9). Among the various types of fractures associated with electrical injuries, spinal fractures exhibit unique characteristics and complexities. In clinical settings, it is essential to recognize that patients with electrical injuries often present with multiple serious comorbidities, which can result in spinal fractures being overlooked, leading to missed or incorrect diagnoses and delaying optimal treatment.

This article presents a case of spinal fracture resulting from electric shock and reviews relevant literature on the topic. The aim is to investigate the pathogenesis, clinical manifestations, diagnostic considerations, and treatment strategies for spinal fractures caused by electric shock. This analysis seeks to provide clinicians with valuable insights and references that enhance the understanding, diagnosis, and management of spinal fractures associated with electrical injuries.

## Case presentation

**Figure 1:**
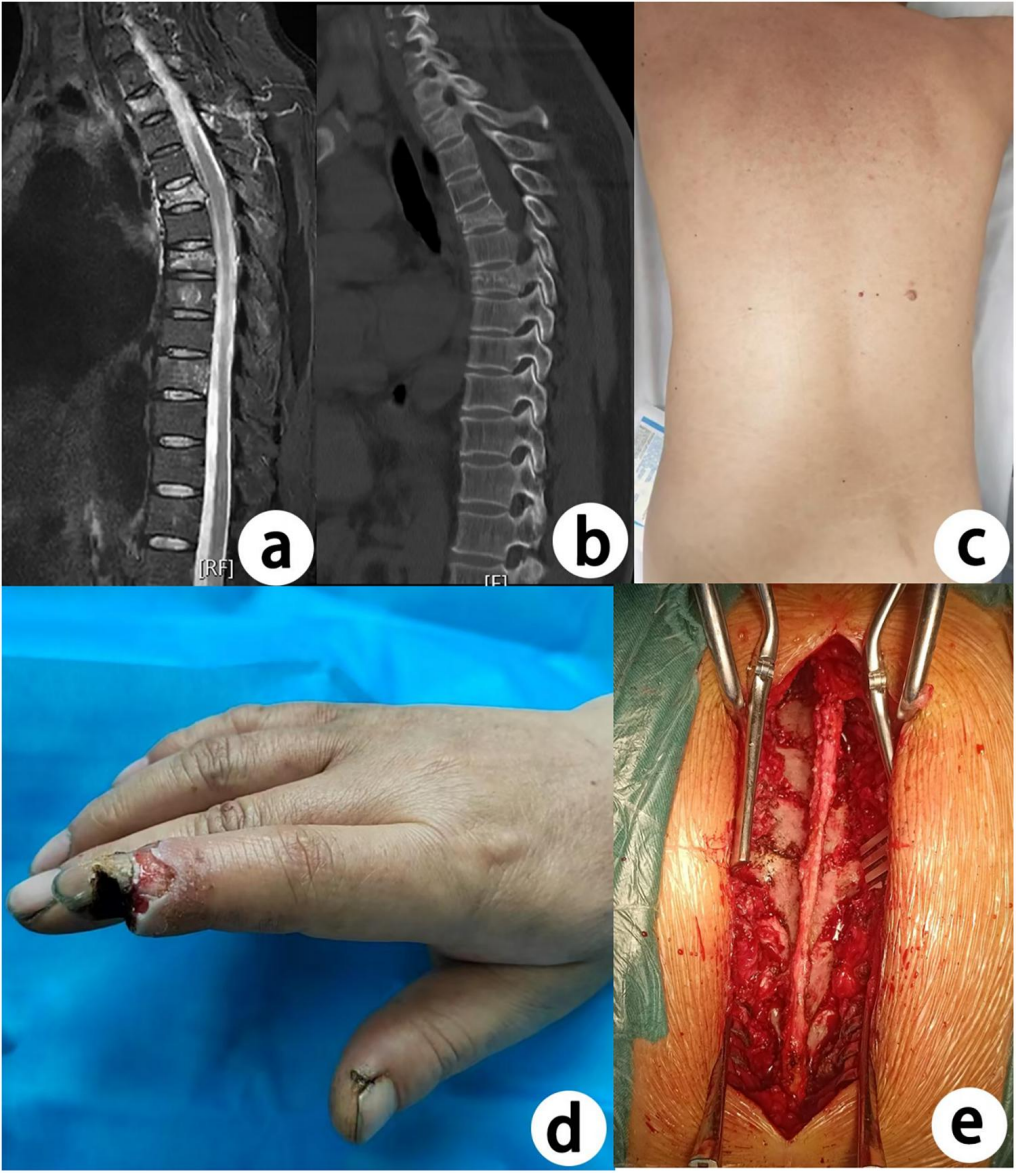
**(a)** preoperative MRI examination revealed high signal intensity in the T1, T4, and T6 vertebrae; **(b)** preoperative CT examination demonstrated fractures of the T4 and T6 vertebrae, along with a collapse of the posterior wall of the vertebrae; **(c)** preoperative appearance of the patient's back: skin intact, no direct traumatic factors; **(d)** electric burns injury on the patient's left hand; **(e)** intraoperative appearance (T3-T7).

A 49-year-old male presented to the Burn Department of our hospital with an electrical injury to his left hand, sustained during occupational exposure to current in a 380 V circuit for 5 seconds, three hours prior to admission. No obvious exit wound was identified on the rest of the body surface. During hospitalization, the patient developed severe thoracolumbar pain, with a visual analog scale (VAS) score of 7. Neurological examination revealed no motor or sensory deficits in the lower extremities. MRI demonstrated abnormal signal intensities at the T1, T4, and T6 vertebral bodies, exhibiting hyperintensity on T2-weighted fat-suppressed sequences and hypointensity on T1-weighted images. Subsequent thoracic computed tomography (CT) revealed retropulsed fracture fragments at the posterior walls of the T4 and T6 vertebrae, encroaching into the spinal canal. Bone mineral density assessment by dual energy x-ray absorptiometry (DXA) indicated severe osteoporosis (T-score = −3.81). The definitive diagnoses were: 1) thoracic burst fractures at T4 and T6; 2) compression fracture at T1. The patient underwent posterior spinal fusion with instrumentation from T3 to T7. Postoperative recovery was uneventful, with remarkable pain relief (VAS decreased to 2). At the three-month follow-up, complete resolution of symptoms was achieved without any complications (Figure 1).

## Systematic review

### Literature search

The electric databases including PubMed, Embase and Web of Science were searched to identify previously reported cases of vertebral fracture caused by electric shock. The publication language was limited in English. The published time were not restricted. The search terms were [“fracture” AND (“lumbar” OR “thoracic” OR “spine” OR “vertebrae”) AND (“electric” OR “electrical”)] by Title/Abstract and/or Topic. In addition, a supplementary search of references cited in all relevant articles was performed. Given the extreme rarity of electrical injury-induced spinal fractures, we included cases caused by accidental electric shock, electroconvulsive therapy, and TASER discharge to maximize the sample size. Studies that did not provide sufficient information for further analysis were excluded (Table 1).

**Table 1:** Characteristics of included cases

Author	Country	Journal	Published year	Case number	Gender	Age (years)	Cause of injury	Accompanying symptoms	Neurological symptoms	Index levels	Treatment options	Clinical outcome
Edward L. Margetts	Kenya	Can Med Assoc J	1956	1	M	49	Electric-Shock Therapy	NO	NO	/	Conservative	/
Giacomoni P	Italy	G Ital Cardiol	1987	1	M	48	Electric-Shock Therapy	/	/	T5-7	Conservative	/
James E. Winslow	America	Ann Emerg Med	2007	1	M	38	Taser X26	NO	NO	T6、8	Conservative	Recovery
Tae Seok Jeong	Korea	UTACD	2017	1	M	50	Electric-Shock Therapy	T = -2.4	NO	T5-8	Conservative	Recovery
Aaron C Tyagi	America	Clin Pract Cases Emerg Med	2017	1	M	23	Taser	NO	NO	T6-8	Conservative	Recovery
Sumit Arora	India	JBJS	2020	1	M	35	Electric-Shock	Burn injurie	NO	L2	PPSF	Recovery
Erik K Koda	America	Am J Case Rep	2020	1	M	46	Electric-Shock Therapy	NO	L1–L2 bilateral sensory neuropathy	L1	Conservative	Referral
David Ritchie	America	Int J Surg Case Rep	2021	1	M	25	Electric-Shock	Bilateral sub-capital femur fractures	NO	T3-7, T9, and T11	Conservative	Recovery
Haydar Sekmen	Turkey	Indian Journal of Surgery	2021	1	M	49	Electric-Shock	Burn injurie	NO	T12	PPSF	Recovery
Jan Žatecký	The Czech Republic	European Journal of Medical Research	2022	1	M	22	Electric-Shock	Burn injurie	NO	T2-6	Conservative	Recovery
Ryosuke Hirota	Japan	Spine Surg Relat Res	2023	1	M	14	Electric-Shock	Burn injurie right hemothorax, left hemothorax, and multiple rib fractures	Complete loss of motor and sensory function caudally from the Th10 level	T7-12	Staged surgery	Recovery
Ruili Jia	China	J Med Case RepJ Med	2024	1	M	37	Electric-Shock	T < -2.5	NO	T9,L5	Conservative	Recovery

Gender: F: Female; M: Male.PPSF:Percutaneous pedicle screwfixation.

### Data extraction

Data extracted from eligible papers included baseline characteristics, neurological examinations, imaging findings, surgical outcomes, etc. Data collection was conducted independently by two reviewers using a standard form. If disagreements persisted, arbitration was conducted by a third review author.

### Quality assessment

The Joanna Briggs Institute (JBI) Case Reports Quality Assessment Tool was used to assess risk of bias in included literature. The quality assessment tool includes 8 points to evaluate the quality of case reports from the aspects of patient history, clinical manifestations, diagnosis, treatment, etc, each item was judged by “yes”, “no”, “unclear” and “not applicable”, and the results were cross-checked by two researchers independently. We determined that articles achieved the adequate standard of quality required for their inclusion if five-eighths or more of the questions were answered “Yes” (Table 2).

**Table 2:** Study quality by the joanna briggs institute (JBI) case reports quality assessment tool.

Author	1	2	3	4	5	6	7	8	Quality Assessment
Edward L. Margetts	Yes	Yes	Yes	Yes	Yes	No	No	Yes	High Quality
Giacomoni P	Yes	Yes	No	Yes	Yes	No	No	Yes	High Quality
James E. Winslow	Yes	Yes	Yes	Yes	Yes	Yes	Yes	Yes	High Quality
Tae Seok Jeong	Yes	Yes	Yes	Yes	Yes	Yes	Yes	Yes	High Quality
Aaron C Tyagi	Yes	Yes	Yes	Yes	Yes	Yes	Yes	Yes	High Quality
Sumit Arora	Yes	Yes	Yes	Yes	Yes	Yes	Yes	Yes	High Quality
Erik K Koda	Yes	Yes	Yes	Yes	Yes	Yes	Yes	Yes	High Quality
David Ritchie	Yes	Yes	Yes	Yes	Yes	Yes	Yes	Yes	High Quality
Haydar Sekmen	Yes	Yes	Yes	Yes	Yes	Yes	Yes	Yes	High Quality
Jan Žatecký	Yes	Yes	Yes	Yes	Yes	Yes	Yes	Yes	High Quality
Ryosuke Hirota	Yes	Yes	Yes	Yes	Yes	Yes	Yes	Yes	High Quality
Ruili Jia	Yes	Yes	Yes	Yes	Yes	Yes	Yes	Yes	High Quality

### Literature selection

**Figure 2:**
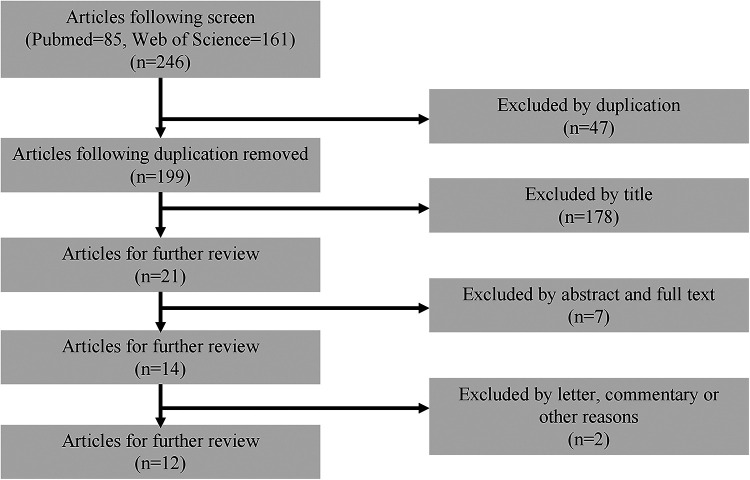
Prisma flowchart of systematic literature review for vertebral fracture caused by electric shock.

A total of 246 documents were retrieved. Following deduplication exclusion, exclusion by titles and abstracts, and exclusion by full texts, a total of 12 documents (6, 10–20) with 12 cases were included in this study. All the studies achieved an adequate level of quality to warrant their inclusion. The detailed search progress was demonstrated by flow chart in Figure 2.

### Data analysis

The baseline characteristics of the included studies were presented in Table 1. Among the 12 cases, all patients were male, with an average age of 36.4 years (ranging from 14 to 50 years). Vertebral fractures resulted from electric shock (*n* = 6), electroconvulsive therapy (*n* = 4), and TASER injury (*n* = 2). Analysis of fracture distribution revealed that 75% (9/12) of compression fractures were predominantly localized to the thoracic spine. Notably, 66.7% (2/3) of patients presented with multiple segment fractures. Surgical interventions included open reduction in three cases. All patients achieved favorable clinical outcomes. Among the 2 patients with baseline neurological deficits, both experienced complete neurological recovery at the final follow-up; no new neurological complications were observed in patients without initial neurological involvement.

## Discussion

This article presents a case who sustained severe spinal fractures following an electric shock injury, despite the absence of any obvious trauma. A literature review revealed that spinal fractures resulting from electric shock injuries are more prevalent in men and frequently involve the thoracic spine. Therefore, in clinical practice, it is essential to consider the possibility of spinal fractures in patients presenting with back pain after experiencing an electric shock injury.

The mechanism of spinal fractures resulting from electrical injury is complex and involves multiple factors, including the thermal effects of electric current, muscle spasms and contractions, as well as secondary blunt trauma (6). When electric current passes through the human body, it generates a significant amount of heat, leading to local tissue burns. Tissues with higher electrical resistance, such as skin, bone, and fat, are more susceptible to heating and solidification (8, 21). This thermal effect not only damages the skin and soft tissues but may also compromise bone stability. Elevated temperatures can lead to the destruction of the bone matrix, rendering the bone structure fragile and more susceptible to fractures (22).

Paraspinal muscle spasms and contractions may play a significant role in vertebral fractures. When an electric current passes through the muscles, it induces spasms and contractions in the multifidus, erector spinae, and psoas muscles. A strong contraction force can subject the vertebral body to considerable stress, potentially resulting in fractures. These muscle spasms not only contribute to localized fractures but may also lead to spinal cord and nerve damage as a secondary consequence of the fracture (10). This mechanism is consistent with the findings of our index case, where severe multiple vertebral fractures occurred despite only 5 seconds of 380 V exposure without significant thermal injury or secondary blunt trauma. It also explains the 75% thoracic spine predominance observed in our systematic review, as the thoracic vertebrae are subjected to the highest biomechanical stress during synchronous paraspinal muscle contractions.

Some vertebral fractures can occur due to blunt trauma, particularly when a patient loses balance as a result of severe muscle spasms and subsequently falls. In such cases, the spine endures direct impact, rendering it vulnerable to fractures. The locations of fractures resulting from this mechanism of injury typically correspond to those caused by falls, predominantly affecting the thoracolumbar region. It is essential to distinguish spinal fractures resulting from this mechanism from those documented in this study.

Bone fragility is an important confounding factor in this case. Severe osteoporosis reduces the mechanical strength of the vertebral body, making it more vulnerable to fracture even under relatively mild stress from muscle spasms induced by electric current. This may explain why this patient developed multiple severe vertebral fractures despite only 5 seconds of exposure to 380 V current. Clinicians should be particularly vigilant about spinal fractures in elderly patients or those with known osteoporosis after electrical injury.

The treatment of electrical injury-induced spinal fractures follows the same principles as traumatic spinal fractures. Conservative management is appropriate for stable fractures without neurological deficits, while surgical intervention is indicated for unstable fractures or those with neurological compromise.

The most important takeaway from this study is that clinicians should maintain a high index of suspicion for spinal fractures in any patient presenting with back pain after electrical injury, even in the absence of a clear history of trauma. Initial assessment of electrical injury patients often focuses on obvious burns and cardiopulmonary complications, which can lead to missed diagnosis of spinal fractures. Routine spinal imaging (preferably MRI) should be considered for all electrical injury patients with back pain, especially male patients and those with osteoporosis.

This study has certain limitations regarding case data and literature review. Firstly, the limitation of case data pertains to the small sample size, as only one patient was reported. This patient had severe osteoporosis, which may influence the occurrence and progression of fractures, potentially undermining the generalizability of the conclusions. Secondly, regarding the literature review, the included studies have varying methodological quality and significant heterogeneity in injury mechanisms. These limitations collectively restrict our comprehensive understanding of this rare condition and hinder the development of standardized clinical management strategies. Future studies should focus on specific electrical injury subtypes (e.g., accidental electric shock, electroconvulsive therapy, TASER injury) to provide more targeted and evidence-based clinical guidance. Thirdly, The male predominance observed in this study is based on a small sample size and cannot be generalized to the entire population. Larger population-based studies are needed to confirm the true gender distribution of this injury.

## Conclusion

All 12 cases included in this systematic review were male patients, which may reflect the higher occupational exposure risk of men to electrical hazards and potential reporting bias. For individuals experiencing back pain following an electric shock injury, particularly male patients, it is essential to consider the potential for spinal fractures to prevent missed diagnoses.

## Data Availability

All relevant data is contained within the article. The original contributions presented in the study are included in the article/Supplementary Material, further inquiries can be directed to the Corresponding Author.
